# Decision-making ability, psychopathology, and brain connectivity

**DOI:** 10.1016/j.neuron.2021.04.019

**Published:** 2021-06-16

**Authors:** Michael Moutoussis, Benjamín Garzón, Sharon Neufeld, Dominik R. Bach, Francesco Rigoli, Ian Goodyer, Edward Bullmore, NSPN Consortium, Peter Fonagy, Peter Jones, Tobias Hauser, Rafael Romero-Garcia, Michelle St Clair, Petra Vértes, Kirstie Whitaker, Becky Inkster, Gita Prabhu, Cinly Ooi, Umar Toseeb, Barry Widmer, Junaid Bhatti, Laura Villis, Ayesha Alrumaithi, Sarah Birt, Aislinn Bowler, Kalia Cleridou, Hina Dadabhoy, Emma Davies, Ashlyn Firkins, Sian Granville, Elizabeth Harding, Alexandra Hopkins, Daniel Isaacs, Janchai King, Danae Kokorikou, Christina Maurice, Cleo McIntosh, Jessica Memarzia, Harriet Mills, Ciara O’Donnell, Sara Pantaleone, Jenny Scott, Pasco Fearon, John Suckling, Anne-Laura van Harmelen, Rogier Kievit, Marc Guitart-Masip, Raymond J. Dolan

**Affiliations:** 1 Wellcome Centre for Human Neuroimaging, University College London, London WC1N 3BG, UK; 2 Max Planck University College London Centre for Computational Psychiatry and Ageing Research, London WC1B 5EH, UK; 3 Aging Research Centre, Karolinska Institute, Stockholm, Sweden; 4 Department of Psychiatry, University of Cambridge, Cambridge CB2 0SZ, UK; 5 Computational Psychiatry Research, Department of Psychiatry, Psychotherapy, and Psychosomatics, Psychiatric Hospital, University of Zurich, 8032 Zurich, Switzerland; 6 Department of Psychology, City University, London, UK

**Keywords:** decision acuity, computational psychiatry, functional connectivity, adolescence, development

## Abstract

Decision-making is a cognitive process of central importance for the quality of our lives. Here, we ask whether a common factor underpins our diverse decision-making abilities. We obtained 32 decision-making measures from 830 young people and identified a common factor that we call “decision acuity,” which was distinct from IQ and reflected a generic decision-making ability. Decision acuity was decreased in those with aberrant thinking and low general social functioning. Crucially, decision acuity and IQ had dissociable brain signatures, in terms of their associated neural networks of resting-state functional connectivity. Decision acuity was reliably measured, and its relationship with functional connectivity was also stable when measured in the same individuals 18 months later. Thus, our behavioral and brain data identify a new cognitive construct that underpins decision-making ability across multiple domains. This construct may be important for understanding mental health, particularly regarding poor social function and aberrant thought patterns.

## Highlights

• Young people have a general decision-making ability, which we call “decision acuity”
• Decision acuity is reflected in how strongly connected certain brain networks are
• Low decision acuity is associated with general social function psychopathology

## Teaser

Moutoussis, Garzón, et al. report that young people have a general decision-making ability, reflected in the functional connectivity of specific brain networks and reduced in those with symptoms of poor general social function. Thus, it may be important for understanding aspects of mental health and its basis in brain function.

## Introduction

Effective decision-making underpins a range of activities that span economic performance and social adaptation. A computational characterization of decision-making processes is also considered important in advancing an understanding of psychiatric disorders (Scholl and Klein-Flügge, 2018). Yet, unlike traditional cognitive constructs such as intelligence, the distribution and covariation of decision-making characteristics in the population is unknown, while the reliability of behavioral tasks typically used to measure these abilities has been questioned (Brown et al., 2020; Enkavi et al., 2019; Hedge et al., 2020). Likewise, although there is a growing knowledge regarding the neural underpinnings of decision-making ability, there is a relative dearth of knowledge in relation to adolescence and early adulthood, a crucial period for brain maturation (Giedd, 2004; Whitaker et al., 2016). Thus, there is an increasing urgency in understanding the neural basis of cognitive development in young people, including its relationship with brain connectivity (Sripada et al., 2020). An added motivation here is the observation that a high proportion of psychopathology emerges during adolescence and early adulthood (Paus et al., 2008).

Decision-making reflects a complex interplay between multiple processes that bear on evaluating options and choosing a course of action. These processes are well characterized within a reinforcement-learning framework (Dolan and Dayan, 2013; Kable and Glimcher, 2009 Phelps et al., 2014; Sutton and Barto, 1998). Here, a distinction is made between a reliance on learning how beneficial an action has been in the past, or alternatively the exploitation of an accurate model of an environment, in order to infer the consequences of each action. Computationally, this encompasses model-free control, accurate model learning (Feher da Silva and Hare, 2020), and model-based evaluation (Daw et al., 2005; Dolan and Dayan, 2013). Model-based and model-free influences trade off at different levels in different individuals (Eppinger et al., 2017; Kool et al., 2017).

A more subtle source of decision variability is the impact of Pavlovian heuristics, reflecting a propensity to attach value to specific actions by mere association with whether they lead to reward or punishment (de Boer et al., 2019; Guitart-Masip et al., 2012; Moutoussis et al., 2018). This conflict is also evident when individuals balance a need to harvest rewards against potential dangers inherent in acting within an uncertain environment (Bach et al., 2020; Loh et al., 2017; O’Neil et al., 2015). This engenders a conflict between motivational drives (e.g., approach versus avoidance) that need resolution in order to enact effective decisions.

There is much variability in decision-making across individuals. One source of this variability pertains to uncertainty in decision outcomes, where a tolerance of uncertainty can drive preferences for risky but, on average, good options (Christopoulos et al., 2009; Payzan-LeNestour et al., 2013). Likewise, individual variability in decision-making is seen also in the temporal domain, where individuals balance exploiting an immediately available safe option against the possibility of greater, possibly uncertain, future benefit (Badre et al., 2012; Sutton and Barto, 1998). Finally, as many decisions are enacted in a social context, understanding the intentions and emotions of others is often crucial for making decisions and impacts on characteristics such as one’s propensity to cooperate with others (Fett et al., 2012; Hula et al., 2015; Luo et al., 2018).

Although the above emphasizes discrete factors as influencing decision-making, we hypothesized that there would also be covariation across decision-making abilities within the population, implying shared variance along latent dimensions. This is analogous to the structure of intelligence, where a cornucopia of abilities co-varies with latent dimensions such as general and domain-specific intelligence (van der Maas et al., 2006). On this basis, we employed a broad-ranging decision-making battery and administered it to 830 14- to 24-year-olds living in the community (Kiddle et al., 2018). The battery included tasks tapping into sensitivity to gains and losses (most tasks in Table 1), the extent to which model-based influences dominate choice evaluation (Table 1, task D but also tasks C, E, and F), a propensity to take risks and exhibit impulsivity (tasks B, C, E, and G), and an ability to make beneficial social judgements (tasks E and F). We hypothesized that these four domains would correspond to latent dimensions of decision-making ability across tasks. We used computational modeling and key descriptive statistics to extract relevant metrics from the tasks (Bach et al., 2020; Fett et al., 2012; Moutoussis et al., 2011, 2016, 2018; Rigoli et al., 2016; Shahar et al., 2019a). Submitting these component metrics to factor analysis (see STAR Methods) allowed us to derive latent across-task cognitive constructs underlying decision-making and test for the presence of latent dimensions corresponding to the hypothesized cognitive domains.

**Table 1:** Decision-making task battery

Task (with key reference)	Broad (selected) psychological domains	Computational constructs assessed	Key individual parameters and descriptive measures
A. Go-NoGo task (Guitart-Masip et al., 2012)	Default (Pavlovian) propensities for action and ability to modify them Impact of gains and losses on choice	Pavlovian biases (i.e., propensity to engage in action in order to obtain rewards and to abstain from action to avoid losses). Reward sensitivity, equivalent to decision temperature. Instrumental learning rate in the appetitive and aversive domains.	1. Pavlovian bias. 2. and 3. Reaction times for action choices in the context of threat versus opportunity. 4. Sensitivity to outcomes. 5. General bias for action rather than non-action. 6. Motivation-independent, “irreducible,” variability in decision-making. 7. and 8. Learning rates in the appetitive and aversive contexts.
B. Economic preferences task (Symmonds et al., 2011) (NB: administered at baseline only)	Risk taking/impulsivity Impact of gains and losses on choice	Baseline taste for gambling. Risk avoidance (preference for outcome distributions of low variance).	9. Overall preference for gambling over known returns. 10. Preference weight for variance, compared to the mean, of an outcome distribution, named “economic risk preference.” 11. Effect of outcome distribution asymmetry (skewness) on preferences. 12. Sensitivity to expected value of outcomes.
C. Approach-avoidance conflict task (Bach et al., 2014)	Risk taking/impulsivity Impact of gains and losses on choice Ability for complex planning	Willingness to expose oneself to different levels of risk for the sake of amassing rewards. Ability to learn about time-dependent hazards and plan efficient sensorimotor sequences to minimize risk.	13.–15. Factor-analytic scores summarizing variance over a comprehensive set of behavioral measures in the task. Approximately corresponding to sensitivity to overall level of threat, sensitivity to the time dependency of threat, and overall performance.
D. Two-step task (Daw et al., 2011)	Ability for complex planning Impact of gains and losses on choice	Strength of “model-free” (i.e., based on directly learned values of actions) versus “model-based” (i.e., explicitly estimating the future consequences of actions) decision-making.	16. Model-basedness: tendency to shift in decisions as a consequence of a different decision being more advantageous according to the transition probabilities inherent in the task. 17. Learning rate. 18. Perseveration tendency. 19. Reward sensitivity. 20. Eligibility trace (propensity of learning to affect not just the current state but also others related to it).
E. Information gathering task (Moutoussis et al., 2011)	Risk taking/impulsivity Ability for complex planning Impact of gains and losses on choice	Assessment of whether future decisions will be more advantageous if one gathers more information.	21. Information sampling noise, which determines not only decision variability but also effective depth of planning. 22. Subjective cost of every piece of information asked for when experimenter imposes no such price explicitly. 23. and 24. Ditto if a fixed, external price-per-step is imposed.
F. Multi-round investor-trustee task (Fett et al., 2012)	Understanding the preferences of others (social cognition) Ability for complex planning Impact of gains and losses on choice	Overall strategies used to elicit cooperation and avoid being exploited by one’s anonymous task partner.	25. Initial trust (i.e., the amount given by the investor to the trustee before they have any specific information about them). 26. Cooperativeness: average degree to which investor and trustee tended to respond to reductions (or increases) in each other’s contributions in kind. 27. Responsiveness: average magnitude of responding to the partner’s change in contribution.
G. Interpersonal-discounting task (Moutoussis et al., 2016)	Understanding the preferences of others (social cognition) Risk taking/impulsivity	Baseline inter-temporal discounting; shift in discounting preferences upon exposure to peers’ preferences.	28. Basic hyperbolic temporal discounting coefficient. 29. Relevance of others’ observed preferences to the self. 30. Discounting taste uncertainty, i.e., uncertainty about one’s own tastes in this domain. 31. Decision variability over choosing for others. 32. Irreducible decision noise.

We assessed construct stability using the data of 571 of our participants who performed the decision-making battery a second time, at a follow-up 18 months later on average, by characterizing the relationship between the inferred latent cognitive constructs and external measures such as age, IQ, and mental health characteristics. Here, we hypothesized that latent dimensions of decision-making would correlate with self-reported psychological dispositions and mental health symptoms. To test this latter hypothesis, we availed participants’ derived scores for both general and specific disposition factors (Polek et al., 2018) as well as concurrent mental health symptoms (St Clair et al., 2017).

Crucially, we characterized the neural circuitry underpinning latent decision-making factors. To achieve this, we analyzed functional connectivity from resting-state functional magnetic resonance imaging (fMRI) data (rsFC), providing a metric of coupling between blood-oxygen-level-dependent (BOLD) time series from different brain regions or networks (nodes). Patterns of rsFC are known to behave as dispositions to a large degree (Finn et al., 2015), including predicting a subject’s cognitive abilities in diverse domains (Dubois et al., 2018; Kong et al., 2019; Rosenberg et al., 2016; Smith et al., 2015). Thus, we could ask whether distinct connectivity networks predicted latent decision-making factors and whether identified connectivity networks had stability over time.

We found evidence for a single dimension of covariation in the population to which multiple decision-making tasks contributed. This dimension, which we termed “decision acuity.” reflected speed of learning, an ability to take account of cognitively distant outcomes, and low decision variability. We found that decision acuity has a reliability that was much higher than that reported for typical decision-making tasks (Moutoussis et al., 2018). In keeping with this, it was associated with distinct patterns of rsFC. Finally, decision acuity was characterized by a functional connectivity signature and a relationship to both psychological dispositions and symptoms that was distinct to that of IQ.

## Results

### Decision acuity is an important dimension of decision-making

A total of 830 young people aged 14–24 were tested using a task battery assessing diverse components of decision-making (Table 1). 349 participants underwent brain fMRI at rest, on the same day as cognitive testing, to assess resting-state functional connectivity profiles. Scanned participants had no history of neuropsychiatric disorder and no suspected psychiatric diagnosis on SCID interview. 50 participants with DSM-5 major depressive disorder were included in the non-scanned sample to compare the structure of their decision-making to the remaining healthy group. The STAR Methods and supplemental information provide further detail on this subgroup.

We extracted 32 decision-making measures from the battery, which we subjected to factor analyses. Exploratory factor analysis was followed by confirmatory analysis and out-of-sample testing of the best factor model (see STAR Methods for details of the factor-analytic approach, including dimensionality estimation and stability analyses).

**Figure 1:**
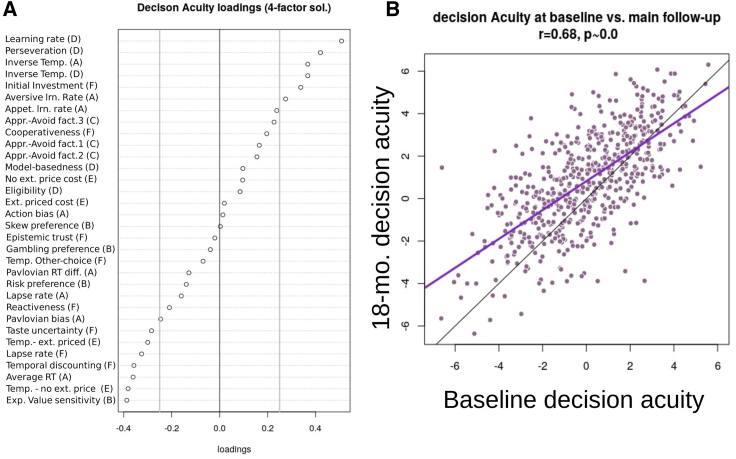
Decision acuity (A) Decision acuity common factor over cognitive parameters, based on the validated four-factor solution. Measure labels are shortened versions of descriptions in Table 1, and letters in brackets are task labels referring to Table 1. The top half of variables load positively, while gray vertical lines give a visual indication of which measures are important, being the thresholds used for inclusion of variables in the confirmatory analyses. (B) Decision acuity was strongly correlated between baseline and follow-up, as expected for a dispositional measure. Mauve is the regression line, and black is the identity line.

Working with the larger, baseline sample, we discerned four stable decision-making factors. Importantly, only the first of these loaded on measures from multiple tasks. We named this factor decision acuity, or *d*, as it loaded negatively on decision variability measures, especially decision temperature, and loaded positively on measures contributing to profitable decision-making, such as low temporal discounting and faster learning rates (Figure 1; Table S1). Thus, participants with high *d* had low decision variability in economic-risk, information-gathering, Go-NoGo, and Two-Step tasks. They had fast reaction times and high learning rates in the Go-NoGo task. Note that a decision temperature parameter can always be re-written as the inverse of reward (and/or loss) sensitivity. Hence, the prominent role of negatively loading temperature parameters in *d* supports our *a priori* hypothesis that reward sensitivity constitutes an important shared characteristic across tasks.

In the baseline sample, we confirmed that *d* correlated with profitable decision-making by estimating a measure of aggregate task performance, based on net points won across tasks and separate from components of *d* (Pearson r = 0.50, p < 1e−10; see supplemental information, part C, for details). Remarkably, *d* predicted this aggregate measure of performance independently from IQ, providing supportive evidence for convergent validity with directly measured task performance. In fact, the effect of IQ on performance depended on its shared variance with *d* (the caveat here being that performance in tasks and *d* share common-method variance).

The other three factors derived from this analysis addressed within-task behavior rather than hypothesized global decision-making constructs and were thus of peripheral interest here. The second selected the delegated inter-temporal discounting task (D), the third the information-gathering task (E), and the fourth the economic risk preference task (C) (Figure S2). As expected, given that each task had a unique focus, constituent cognitive measures showed high uniqueness scores across all factors. 22 of the 32 measures had uniqueness > 80% (Figure 1B).

### Developmental features of decision acuity

We first examined how *d* depended on age, a key indicator of development. We used linear mixed effects (LME) analysis with participant as random effect, two measurement time points of decision acuity and IQ, and one (baseline) score per participant for dispositions, self-reported sex and socioeconomic variables. LME analysis modeled age both longitudinally and cross-sectionally. This analysis showed that the *d* varied in the same manner with age within or across participants (beta = 0.24, SE = 0.022, p ∼ 0.0 [undetectable]), suggesting that *d* increased with development. *d* was stable from baseline to follow-up, although slightly less so than IQ was (Wechsler Abbreviated Scale of Intelligence, WASI) (r = 0.68, p ∼ 0.0 for *d*; 0.77, p ∼ 0.0 for WASI IQ; 95% CI for the difference = −0.135 to −0.044; Figure 1B). These estimates give a conservative estimate of discriminant validity of *d* versus IQ D = 0.76, which is satisfactory (<0.85) (Voorhees et al., 2016). *d* increased with testing wave (effect size = 0.38, p ∼ 0.0). We found no evidence here, or in subsequent analyses, for more complex models of age (curvilinear effects or interactions with sex).

We confirmed that both matrix and vocabulary raw IQ subscores robustly correlated with *d* (fixed-effect betas = 0.088, 0.179, SE = 0.008, 0.018, p ∼ 0.0). However, inclusion of raw IQ scores did not affect the significance of age as a regressor (age beta = 0.121, SE = 0.020, p ∼ 0.0). Therefore, not only did decision acuity increase with age in our sample but so did the component that was independent of IQ abilities, suggesting that IQ and *d* developed in parallel with age. Together, IQ subscores and age accounted for r^2^_adj_ = 0.31 of the variance in *d* at baseline.

With respect to self-reported sex, *d* scores for males were higher than those of females at baseline (t test p = 8.6e–5, effect size = 0.27). However, if both IQ subscores and age were entered in LME, the correlation between *d* and self-reported sex was no longer significant. Thus, any uncorrected sex dependence is likely to be due to participant self-selection. That is, among males, more participants of higher IQ volunteered relative to among females. *d* showed no significant age × sex dependence (controlling for IQ, sex p = 0.39, age × sex p = 0.21).

As to socioeconomic factors affecting the development of *d,* we noted an increase with parental education (p = 0.0051, beta = 0.19, SE = 0.067) but no significant association with neighborhood deprivation (p = 0.09).

### Mental health factors and their association with decision acuity

Next, we examined the relationship between *d* and both psychological dispositions and symptoms. Note that in our study, involving mainly healthy adolescents and young adults, symptomatology refers to the nature and extent of self-reported mental health symptoms rather than diagnosable clinical disorders. Thus, we used factor scores validated specifically for our sample (Polek et al., 2018; St Clair et al., 2017), which indicated that dispositions and symptoms in our sample were well described by bifactor models. Each bifactor model comprises a superordinate “general factor” and subordinate “specific factors.” Dispositions comprise a general social functioning factor (“sociality”) and four specific factors: social sensitivity, sensation seeking, effortful control, and suspiciousness. Symptoms comprise a general distress factor, a.k.a. “p factor” (Caspi et al., 2014; Patalay et al., 2015), and five specific factors: mood, self-confidence, worry, aberrant thinking, and antisocial behavior.

*d* was significantly predicted by dispositions, over and above its relationship with intelligence. We first regressed all symptom disposition factor scores against *d*, allowing all factors to compete in explaining variance in LME models with participant intercept as random effect. *d* was significantly and positively related to the general disposition factor, sociality (p = 0.0002, standardized beta, a.k.a. bz = 0.36, SE(bz) = 0.096). In models that included raw IQ scores and age, both variables significantly predicted *d* and improved model fit (Baysian Information Criterion, a.k.a BIC = 4,873 versus 5,083 without IQ). Importantly, inclusion of IQ strengthened the significance of sociality (p = 0.0001, bz = 0.32, SE(bz) = 0.084; see Table 2).

**Table 2:** Key steps in regression analyses

Independent variable	A. Symptoms only (p value for fixed effects beta; time-dependent LME)	B. Dispositions only (p value for beta; baseline only)	C. Symptoms and dispositions (p value for fixed effects beta; time-dependent LME)
General symptom factor: General distress	0.048∗	–	0.390
Self-confidence specific factor (SF)	0.351	–	0.316
Antisocial behavior SF	0.381	–	0.912
Worry SF	0.014∗	–	0.875
Aberrant thinking SF	0.016∗	–	0.074#
Mood SF	0.813	–	0.871
General disposition factor: Adaptive sociality	–	0.0018∗∗	0.0001∗∗∗
Social sensitivity	–	0.656	–
Sensation seeking	–	0.987	–
Effortful control	–	0.959	–
Suspiciousness	–	0.014∗	–
Age	<0.0001∗∗∗	0.0002∗∗∗	<0.0001∗∗∗
Vocabulary IQ (raw score)	<0.0001∗∗∗	<0.0001∗∗∗	<0.0001∗∗∗
Matrix IQ (raw score)	<0.0001∗∗∗	<0.0001∗∗∗	<0.0001∗∗∗

∗ significant at p = 0.05.

∗∗ significant at p = 0.005.

∗∗∗ significant at p < 0.001.

# trend level significance at p = 0.05.

Among symptom scores, *d* was most strongly associated with aberrant thinking, which draws on schizotypy and obsessionality. Covarying for IQ, but not dispositions, showed that *d* significantly decreased with higher aberrant thinking (p = 0.016, beta = −0.16, SE = 0.066), higher general distress (p = −0.048, beta = −0.12, SE = 0.057), but lower worry (p = 0.014, beta = 0.16, SE = 0.063). However, covarying for sociality (with or without other dispositions) reduced the significance of aberrant thinking, to trend level (p = 0.074, beta = −0.10, SE = 0.053), and abolished the relationship with other symptom dimensions (symptom general factor: distress, p = 0.82, others ranging from p = 0.35 to 0.99). By itself, IQ was significantly correlated to aberrant thinking (matrix p = 0.013, vocabulary p = 0.0001) and less so general distress (matrix p = 0.012, vocabulary p = 0.47). Again, all analyses linearly accounted for age and did not benefit from more complex models of age.

### Patterns of brain connectivity are associated with decision acuity differently from IQ

**Figure 2:**
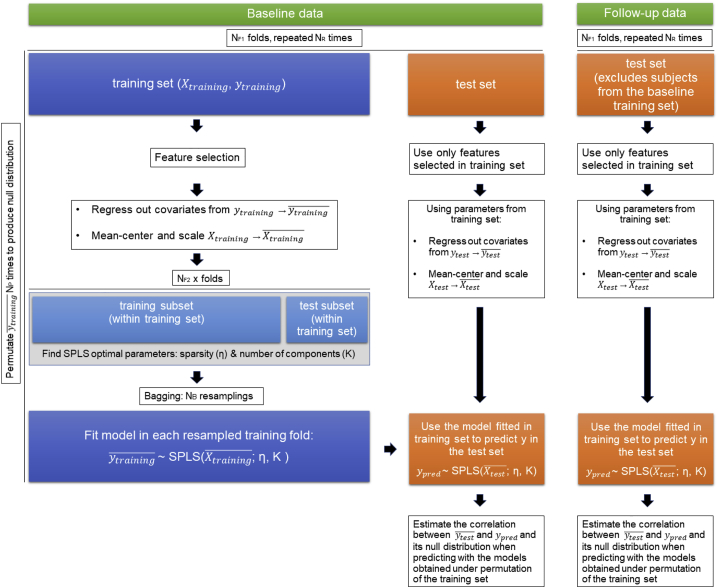
Structure of predictive testing Flow diagram of the nested cross-validation pipeline used to estimate how strongly decision acuity (similarly for IQ) could be predicted from brain data. Essentially, a predictive model was derived from training folds and then applied to the brain data from test folds to derive predicted values for the decision acuity for each individual. This could then be compared with the experimentally derived decision acuity. In our study, N_B_ = 200, N_F1_ = 20, N_F2_ = 10, N_R_ = 5, and N_P_ = 100. *X* corresponds to the rsFC features and *y* to the scores predicted (*d* or IQ).

Out of 313 healthy subjects who were scanned at baseline, we discarded baseline scans without acceptable imaging data quality (3), whose ME-ICA denoising did not converge (4), or who had excessive motion while scanning (8), leaving 298 baseline scans for analysis. A further three subjects were removed from analyses involving IQ scores as they did not complete the IQ tests, leaving 295 subjects for analysis. A population-average parcellation of brain data was obtained using independent component analysis in our sample, resulting in 168 networks (nodes) *within* each of which activity was highly correlated. Patterns of connectivity *between* nodes were then estimated as partial correlation values, or resting-state functional connectivity (rsFC). We then used rsFC values as features in sparse partial least-squares (SPLS) analyses to predict decision acuity and composite IQ. We used cross-validation and out-of-sample predictive testing to prevent overfitting. Predictive accuracy was assessed as Pearson’s correlation coefficient between true scores and model-predicted values. We report associations between predicted and observed decision acuity after correcting for scanner-related and other covariates. This ensures that it is the information carried by the functional connectivity alone that predicts cognitive abilities. (See STAR Methods for details; Figure 2 illustrates the structure of the predictive testing.)

Scores for *d* predicted on the basis of functional connectivity, *d*_*pr*_, significantly correlated with measured *d* controlling for demographic and imaging-related covariates (see STAR Methods for details; r = 0.145, p < 10^−6^). The correlation between measured IQ and IQ predicted on the basis of rsFC using all connections was lower but also significant (r = 0.092, p = 9e–5).

To interpret the neuroanatomical structure of the predictive model, we first partitioned the nodes into anatomically meaningful “modules” using a community detection algorithm (Blondel et al., 2008) and then asked how well each of these modules predicted *d*. The community detection algorithm clustered the nodes into disjoint communities or modules based on the strength of their intrinsic connectivity, to some extent analogous to large-scale functional networks. As shown in Figure 2, we obtained the following modules: anterior temporal cortex including the medial temporal lobe (ATC); frontal pole (FPL); frontoparietal control network (FPN); left dorsolateral prefrontal cortex (LDC); medial prefrontal cortex (MPC); orbitofrontal cortex, medial and lateral (OFC); opercular cortex (OPC); posterior cingulate cortex (PCC); posterior temporal cortex (PTC); right dorsolateral prefrontal cortex (RDC); subcortical (SUB); salience network (SAN); somatosensory and motor areas (SMT); and visual regions (VIS). We fitted a different SPLS model to the subset of connections involving the nodes in each module, including both intra- and intermodular connections.

The correlation between measured and predicted *d* scores was significant for the FPN, MPC, OFC, OPC, PCC, SMT, and VIS modules after correction for multiple tests (Figure 4A; Table 2), with the strongest correlations for OFC, PCC, and SMT. For the PCC and SMT modules, the correlation coefficients exceeded to a small degree the correlation for a model employing all possible connections. This can best be explained as a result of feature selection. In the full model, it is harder to select just the right features and protect against over-fitting, resulting in a greater penalty in predictive accuracy. On the other hand, the model trained on a smaller set of features alone is less likely to overfit. This paradoxical increase in accuracy for a model with less features is known to be stronger when the number of observations is small, relative to the number of features (Chu et al., 2012), which is the case in our dataset. The different modules comprised diverse numbers of nodes, but there was no significant association between the number of model features and the correlation between observed and predicted scores (*d*: r = 0.356, p = 0.193; IQ composite scores: r = −0.158, p = 0.574).

**Figure 3:**
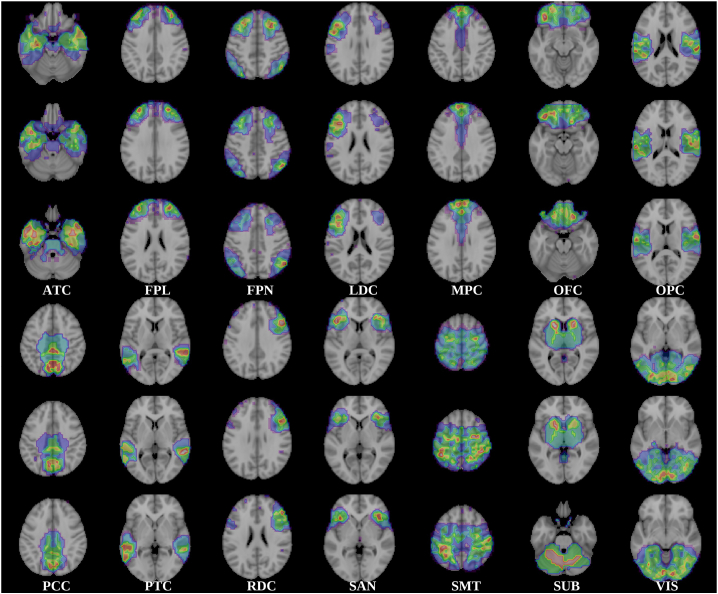
Brain networks Modules detected by the community structure algorithm. The 168 nodes of the parcellation were clustered in 14 modules with high average rsFC among their nodes. ATC, anterior temporal cortex including the medial temporal lobe; FPL, frontal pole; FPN, frontoparietal control network; LDC, left dorsolateral prefrontal cortex; MPC, medial prefrontal cortex; OFC, orbitofrontal cortex, medial and lateral; OPC, opercular cortex; PCC, posterior cingulate cortex; PTC, posterior temporal cortex; RDC, right dorsolateral prefrontal cortex; SUB, subcortical; SAN, salience network; SMT, somatosensory and motor areas; VIS, visual regions.

**Figure 4:**
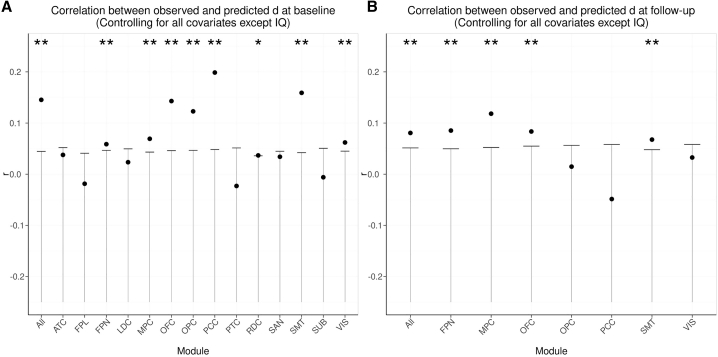
Observed versus predicted decision acuity by testing wave Model predictive performance for each of the functional modules. (A) Coefficient for the correlation between observed *d* and *d*_*pr*_ predicted by models trained on all connections and the connections involving nodes in each module. (B) Correlation between observed *d* and *d*_*pr*_ predicted by models trained on the baseline data. Only modules for which the prediction was significant at baseline are shown here. All the models included as covariates demographic and imaging-related factors (brain volume, scanning site, head motion; see STAR Methods). The whiskers indicate the intervals containing the lower 95% probability mass (corresponding to one-tailed tests) for the null distribution, obtained via permutation of the subjects to derive the significance of the correlation between predicted and measured scores (see STAR Methods). The correlation is significant (uncorrected) when it falls above the whisker. ^∗^significant uncorrected; ^∗∗^significant with FDR correction for the 15 tests. ATC, anterior temporal cortex including the medial temporal lobe; FPL, frontal pole; FPN, frontoparietal control network; LDC, left dorsolateral prefrontal cortex; MPC, medial prefrontal cortex; OFC, orbitofrontal cortex, medial and lateral; OPC, opercular cortex; PCC, posterior cingulate cortex; PTC, posterior temporal cortex; RDC, right dorsolateral prefrontal cortex; SUB, subcortical; SAN, salience network; SMT, somatosensory and motor areas; VIS, visual regions.

Out of 235 subjects who were scanned at follow-up, adhering to the same criteria as for the baseline data, we discarded those without acceptable imaging data quality (4), whose ME-ICA denoising did not converge (5), and who presented with excessive motion (3), leaving 223 subjects available for analysis. We applied the model trained on the baseline data to the follow-up data (see STAR Methods) for the modules where the prediction was significant at baseline. Importantly, the prediction of a subject at follow-up did not involve their own rsFC baseline data, as this would inflate the estimate of predictive performance. The baseline model predicted significantly the follow-up *d* values based on the follow-up connectivity data when using either all the connections or those with networks in the FPN, MPC, OFC, and SMT modules, controlling for demographic and imaging-related covariates, and correcting for multiple tests (Figure 4B; Table 3).

**Table 3:** Correlation coefficients between observed and predicted scores

	Prediction of *d* at baseline (Figure 3A)			Prediction of *d* at follow-up (Figure 3B)			Prediction of *d* at baseline controlling for IQ (Figure 3C)			Prediction of IQ at baseline controlling for *d* (Figure 3D)		
Network	r	p value	p value (FDR corr.)	r	p value	p value (FDR corr.)	r	p value	p value (FDR corr.)	r	p value	p value (FDR corr.)
All	0.145	<1e–6	<1e–6^∗∗^	0.081	0.005	0.018^∗∗^	0.021	0.241	0.651	−0.054	0.972	1.000
ATC	0.038	0.116	0.158	0.052	0.048	0.102^∗^	0.018	0.304	0.651	−0.169	1.000	1.000
FPL	−0.019	0.773	0.773	0.023	0.242	0.363	−0.016	0.712	1.000	0.036	0.130	0.488
FPN	0.059	0.019	0.036^∗∗^	0.085	0.002	0.012^∗∗^	−0.007	0.605	1.000	−0.045	0.979	1.000
LDC	0.023	0.218	0.273	−0.055	0.943	0.985	−0.051	0.950	1.000	0.069	0.015	0.073^∗^
MPC	0.069	0.004	0.011^∗∗^	0.118	9.38e–05	7.03e–04^∗∗^	0.017	0.268	0.651	−0.052	0.960	1.000
OFC	0.143	<1e–6	<1e–6^∗∗^	0.083	0.006	0.018^∗∗^	0.032	0.153	0.574	0.013	0.320	0.960
OPC	0.123	6.79e–06	2.04e–05^∗∗^	0.015	0.333	0.455	0.181	<1e–6	<1e–6^∗∗^	0.170	<1e–6	<1e–6^∗∗^
PCC	0.199	<1e–6	<1e–6^∗∗^	−0.049	0.915	0.985	0.104	2.11e–04	0.001^∗∗^	−0.044	0.955	1.000
PTC	−0.023	0.769	0.773	0.167	<1e–6	3e–06^∗∗^	−0.035	0.877	1.000	0.113	7.2e–05	5.4e–04^∗∗^
RDC	0.037	0.047	0.078^∗^	−0.072	0.985	0.985	−0.101	1.000	1.000	−0.019	0.727	1.000
SAN	0.034	0.106	0.158	0.004	0.448	0.560	−0.138	1.000	1.000	−0.103	1.000	1.000
SMT	0.159	<1ev6	<1e–6^∗∗^	0.068	0.010	0.025^∗∗^	0.107	2.77e–05	2.07e–04^∗∗^	−0.095	1.000	1.000
SUB	−0.006	0.577	0.666	0.022	0.229	0.363	−0.020	0.774	1.000	−0.061	0.980	1.000
VIS	0.062	0.012	0.025^∗∗^	0.033	0.178	0.334	−0.078	0.998	1.000	−0.008	0.606	1.000

**Figure 5:**
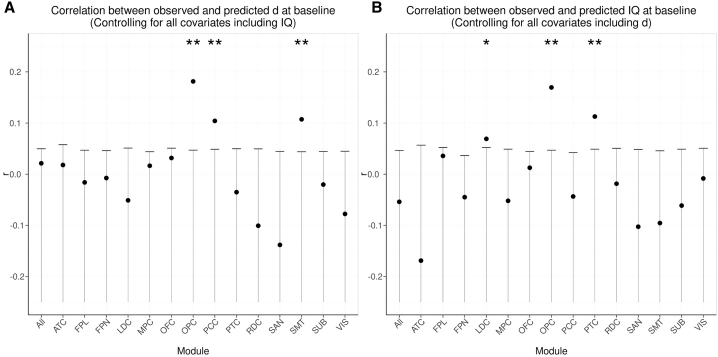
Networks specific to decision acuity versus specific to IQ Predictive performance for *d* and IQ when correcting for each other. (A) As in Figure 4A, correlation between observed *d* and *d*_*pr*_, but here additionally correcting for IQ in addition to demographic and imaging-related factors (brain volume, scanning site, head motion; see STAR Methods). (B) Correlation between observed and predicted IQ, but correcting for imaging related factors and decision acuity. In all plots, the leftmost bar corresponds to the model that includes all connections. The whiskers indicate the intervals containing the lower 95% probability mass (corresponding to one-tailed tests) for the null distribution, obtained via permutation of the subjects to derive the significance of the correlation between predicted and measured scores (see STAR Methods). The correlation is significant (uncorrected) when it falls above the whisker. ^∗^significant uncorrected; ^∗∗^significant with FDR correction for the 15 tests. Abbreviations as per Figure 4.

Correlation coefficients corresponding to the plots in Figures 4 and 5. ^∗^significant uncorrected; ^∗∗^significant with FDR correction for the 15 tests.

To assess whether *d* and IQ can be predicted by specific rsFC patterns or, alternatively, whether both are underpinned by similar patterns of neural connectivity, we controlled the partial correlation coefficients between *d*_*pr*_ and *d*, on top of the nuisance covariates previously included, for IQ. In a complementary manner, we controlled the partial correlation between *IQ*_*pr*_ and *IQ*, on top of the nuisance covariates, for *d*. After correction for IQ composite scores, and correcting for multiple comparisons, the correlation between *d* and *d*_*pr*_ remained significant for OPC, PCC, and SMT (Figure 5A; Table 2), suggesting that these modules reflect decision acuity over and above their relation to IQ. On the other hand, the correlation between IQ_*pr*_ and IQ was significant for OPC and PTC after controlling for *d* (Figure 5B; Table 3), suggesting that these modules reflect IQ over and above their relation to decision acuity. These analyses demonstrate that decision acuity and IQ have distinguishable and specific signatures in functional connectivity networks: decision acuity taps on the default mode, salience, and sensorimotor networks, whereas IQ taps on the salience network but also on temporal networks associated with language processing.

## Discussion

To our knowledge, this is the first study characterizing a dimensional structure in core decision-making from an epidemiologically informed sample of adolescents and young adults. We found that decision-making performance could be described by a broad construct receiving contributions from multiple domains of cognition. We termed this decision acuity, *d*. In our sample, *d* showed satisfactory longitudinal stability, increased with age and with IQ. *d* also had specific associations with mental health measures, over and above IQ. Decision acuity was related to brain function, showing a temporally stable association with rsFC, involving networks previously implicated in decision-making processes. Moreover, rsFC patterns associated with *d* and IQ were distinguishable and specific despite showing a degree of overlap.

Decision acuity had an interpretable structure, reflecting a facility for good decision-making. Decision acuity increased as decision variability lessened, evidenced by its loadings on decision-noise-like parameters across all tasks that provided such measures. The most prominent loadings were inverse temperature parameters, also known as reward sensitivities. By definition, high temperature (a.k.a. reduced reward sensitivity) agents are less motivated about relevant outcomes, supporting our hypothesis that reward sensitivity loaded on an important common factor. However, *d* also received substantial contributions from measures that did not directly reflect reward sensitivity but characterized good decision-making. These included low temporal discounting, fast reaction times, high learning rates, baseline trust in others, low propensity for retaliation, low propensity to show a Pavlovian bias, and low lapse rates. Such non-temperature constructs may also be linked to decision variability, albeit less directly.

An interesting interpretation of this pattern is that lower-acuity participants may find it too costly to eliminate computational errors in the fast pace of many tasks. For example, the computations required to make decisions about outcomes far in the future may be hard to perform for low-*d* agents, resulting in discounting-like behavior. Lapse rates may be understood as “floor” error rates imposed by computational costs. That is, decision-making independent of the value of outcomes may take place when these values are too difficult to compute. Higher decision variability may also be driven by effective beliefs about the world, for example, a belief that overvalues exploration. If working out the correct action is too difficult, trial and error is a brute-force alternative, providing a compensatory or adaptation strategy in the face of limited cognitive resources. Overall, the contrast of noise with precision-enhancing measures in this factor is reminiscent of the association between low ability to reach goals and low policy confidence in active inference (Friston et al., 2013). The agnostic derivation but interpretable nature of *d* can thus be seen as an example of data-driven ontology (Eisenberg et al., 2019).

One remarkable result of our study is the relatively high reliability of our new construct. This is important because many behavioral tasks have low test-retest reliability (Enkavi et al., 2019), an issue that also applies to some of the decision-making measures used in our battery (Moutoussis et al., 2018; Shahar et al., 2019a). A discordance in reliability between the individual decision-making tasks and our decision acuity construct is likely to stem from the fact that the latter reflects shared variance across multiple independent measures. Similarly, self-report surveys previously shown to have high reliability typically involve multiple questions to assess underlying constructs (Enkavi et al., 2019), suggesting that, to obtain reliable decision-making measures, it is useful to use multiple tasks. *d* also showed satisfactory discriminant validity with respect to IQ, which is evidence that it provides distinct meaningful information. Nonetheless, it would be advantageous if individual decision-making measures were refined to improve their reliability and construct validity, and an important example here relates to the task assessing model-basedness (Feher da Silva and Hare, 2020; Kool et al., 2017).

Developmentally, high decision acuity was robustly associated with age, increasing by 0.37 SD from age 14 to 24. This is important because component parameters have been found to have less robust relationships with age in this same sample (Moutoussis et al., 2016, 2018). That *d* varied similarly with age across and within participants offers some reassurance that its age dependence here is not a practice effect. *d* also increased with parental education, a developmentally important socio-economic indicator (McDermott et al., 2019), again independently of IQ. These positive associations of *d* may reflect adolescents and younger adults getting more confident in the outcomes of their actions as a function of maturation but also of a supportive environment.

Mental health indices were associated with *d*, over and above IQ. *d* decreased with p factor (general distress factor) and an aberrant thinking (schizotypy/obsessionality) specific factor and increased with a worry-specific factor but, perhaps counterintuitively, was not associated with the mood-specific factor. *d* explained a small proportion of the variance in mental health, as is often the case for risk factors in community samples like ours (Pearson et al., 2015). Importantly, *d* was most strongly associated with the general disposition factor sociality, which statistically explained most of the relation between *d* and symptoms. Our finding that participants with lower decision acuity had higher residual symptoms (i.e., unrelated to general distress) within the domain of aberrant thinking is consistent with existing literature (Ettinger et al., 2015).

Future mental health research can build on our evidence that decision acuity may reflect a risk factor for schizotypy/compulsivity/obsessionality (aberrant thinking) and general distress (p factor). Thus, decision acuity may confer (or indicate) vulnerability to specific psychopathologies. At the same time, we found that low decision acuity was robustly associated with poor social functioning. Further research is needed to trace the pathways between decision acuity, adaptive social function, and psychiatric symptoms, especially as poor social functioning may confer a greater functional impact to psychiatric symptoms. Finally, a weak relationship with common mental disorder symptomatology, such as anxiety and depression, was a surprise and provides a challenge for the enterprise of identifying computational phenotypes. Replicating these results and establishing their causes beyond the goals of our study can provide new research directions for computational psychiatry, and this dovetails with recent work in related fields (Chen et al., 2020; Sripada et al., 2020).

Decision acuity was also associated with specific, distributed patterns of resting-state brain connectivity (Dubois et al., 2018; Smith et al., 2015). The whole-brain, connectivity-based predictive model depended on connections spread across the entire brain, implying that *d*, like IQ, depends on more extensive systems than those typically observed for state-tapping tasks in functional imaging studies (e.g., medial prefrontal, dorsolateral prefrontal). Strikingly, the pattern of connections predicting *d* was structured, with connections involving nodes in FPN, MPC, OFC, OPC, PCC, SMT, and VIS being most predictive of *d*, irrespective of age and sex. Furthermore, the models trained at baseline on all the features, as well as those restricted on features within FPN, MPC, OFC, and SMT, were also predictive of *d* at follow-up, demonstrating the stability of the relationship between rsFC in these modules and *d* over time.

Reassuringly, decision acuity was predicted by connections involving MPC and OFC, regions typically recruited by decision-making tasks. Circuits involving these regions receive highly processed sensory information and support instrumental behavior by representing subjective value of stimuli and choices (Garvert et al., 2015; Padoa-Schioppa and Assad, 2006; Rushworth et al., 2011). The OFC also supports credit assignment during reward learning (Jocham et al., 2016; Walton et al., 2010) probably by representing an association between stimuli and outcomes (Boorman et al., 2016; Padoa-Schioppa and Assad, 2006; Stalnaker et al., 2018). Finally, the OFC has also been suggested to support the representation of latent states necessary to navigate decision-making tasks (Schuck et al., 2016; Wilson et al., 2014). Similarly, involvement of the PCC, FPN, and SMT is not surprising. Activity in the PCC has been observed during decision-making tasks, and it has been suggested that the PCC monitors the environment to detect transitions to new states (Pearson et al., 2011). Although the frontoparietal circuit has mainly been associated with working-memory task performance (Murray et al., 2017), it has been shown that working memory also contributes to learning in typical reinforcement learning tasks (Collins et al., 2017; Collins and Frank, 2018). Finally, connections involving motor and somatosensory areas may contribute to adaptive decision-making. For example, in our tasks, motor actions were orthogonalized with respect to choices, and recent work suggests that only the more capable decision-makers successfully uncouple motor action and option choice (Shahar et al., 2019b). Hence, SMT connectivity may be important to realize this decoupling. Similarly, active suppression of Pavlovian tendencies that can corrupt optimal decision-making may also involve optimal sensorimotor functioning (Cavanagh et al., 2013; Swart et al., 2018).

Our ability to predict decision acuity at baseline when controlling for IQ, and IQ when controlling for decision acuity, based on particular connectivity modules supports the idea that both constructs have specific signatures in rsFC. This suggests that decision acuity has a neurobiological substrate distinct from that of IQ and adds to the validation of their distinctiveness suggested by their differential association with psychological measures. Although IQ absorbed the predictive ability of the connections within the FPN, the MPC, and OFC, decision acuity tapped on modules within the default mode (PCC), opercular (OPC), and sensorimotor (SMT) networks independently of IQ. On the other hand, IQ tapped on the opercular network (OPC), too, but also on temporal networks associated with language processing (PTC), consistent with the vocabulary subscale of IQ being heavily reliant on linguistic ability (Axelrod, 2002). Interestingly, connections within the OPC, which encompasses the insula, independently contributed to predicting both decision acuity and IQ at baseline. As part of a salience network, these regions may contribute to modulation of the switching between internally and externally directed cognitions (Uddin, 2015).

Important questions for future research include whether decision acuity is a superordinate latent trait of decision-making and whether it relates to dimensions such as risk preference, model-based choice, and aspects of social competence. Crucially, studies informed by the associations found here (aberrant thinking, sociality) can be extended to clinical populations to assess the generality of the findings, as well as to determine whether decision acuity might inform diagnosis and treatment plans for individual psychiatric patients. Such clinical studies can profit from our finding that rsFC can predict (estimate) decision acuity, particularly as rsFC data can be acquired quickly, does not impose cognitive demands, and can be administered repeatedly to characterize patients through different phases of a disorder. This type of extension of our approach will benefit from advances in computational modeling of cognitive and behavioral data (Huys et al., 2016), as well as improvements in imaging data collection, processing, and characterization (Ciric et al., 2018; Kundu et al., 2017; Todd et al., 2016; Vidaurre et al., 2017), including initiatives to acquire high-quality large-scale datasets (Kiddle et al., 2018; Van Essen et al., 2013).

We acknowledge limitations of the present study. We had a retention rate between baseline and follow-up of 70%. Although this is acceptable, it meant that our follow-up sample was smaller, and we had reduced power to detect longitudinal effects. Although epidemiologically stratified, our sample was a volunteer one, introducing potential self-selection biases. Our sample did not allow for many-way (cognitive-brain-developmental-clinical) analyses. Finally, the reliability and ecological validity of task-based measures would benefit from further improvement.

### Conclusion

We describe a new cognitive construct—decision acuity—that captures global decision-making ability. High decision acuity prominently reflected low decision variability. Decision acuity showed acceptable reliability, increased with age, and was associated with mental health symptoms independently of intelligence. Crucially, it was associated with distinctive resting-state networks, in particular in brain regions typically engaged by decision-making tasks. The association between decision acuity and functional connectivity was temporally stable and distinct from that of IQ.

## STAR★Methods

### Key resources table

**Table:** 

REAGENT or RESOURCE	SOURCE	IDENTIFIER
**Deposited data**		

Processed connectivity matrices	This paper	https://github.com/benjamingarzon/FCPC/tree/master/data
ICA maps and functional modules	This paper	https://github.com/benjamingarzon/FCPC/tree/master/data
Data for cognitive task factor analyses	This paper	https://github.com/mmoutou/decAc file AllD18.R
Data for Decision Acuity londitudinal analyses	This paper	https://github.com/mmoutou/decAc file symfacdeciq.csv
Scripts for cognitive task factor analyses and Decision Acuity longitudinal analyze	This paper	https://github.com/mmoutou/decAc R files CFA-decAc.R and decAclongi.R; optional utilities file gen_ut.R

**Software and algorithms**		

MATLAB	Mathworks	RRID: SCR_001622; https://www.mathworks.com/
R package	The R Foundation	RRID: SCR_001905; https://www.r-project.org
ME-ICA	Kundu et al., 2017	https://afni.nimh.nih.gov/pub/dist/src/pkundu/README.meica
FSL	Smith et al., 2004	RRID: SCR_002823; https://fsl.fmrib.ox.ac.uk/fsl/fslwiki/
SPLS R library	Chun and Keleş, 2010	https://cran.r-project.org/web/packages/spls
Brain Connectivity Toolbox	Rubinov and Sporns, 2010	RRID: SCR_004841; http://www.brain-connectivity-toolbox.net
Functional connectivity analysis scripts	This paper	https://github.com/benjamingarzon/FCPC

### Resource availability

#### Lead contact

Further information and requests for resources should be directed to and will be fulfilled by the Lead Contact, Michael Moutoussis (m.moutoussis@ucl.ac.uk).

#### Materials availability

Not applicable

#### Data and code availability

Due to the wording of the consent that participants gave to the NSPN project, all pseudo-anonymized data supporting this study is available upon legitimate-interest request from openNSPN@medschl.cam.ac.uk.

All code pertaining to the analysis of computational task measures and their factor analysis is available upon request from m.moutoussis@ucl.ac.uk, while code pertaining to the functional connectivity analysis is available upon request from benjamin.garzon@ki.se.

### Experimental model and subjects details

#### Human subjects

Participants were sampled from a pool of c. 2400 community-dwelling young people and formed a ‘cognitive cohort’. Participants were contacted at random from 5 age bins (14-16,16-18 etc.), until each recruited age bin had approximately equal proportions of females and males. The proportion of non-white-English youngsters in our study was within 10% of that of the most recent census. Significant neuropsychiatric problems were screened out by self-report, and recruitment sources were selected for the sample to be as representative as possible of the healthy population (Kiddle et al., 2018). We continued to invite people from the larger pool into the cognitive cohort, until our target number of 780 ‘cognitive’ participants was completed. Of these, 300 were invited for MRI brain scanning. They were equally distributed in the 5 age bins as above, and equal Female:Male ratio. At the time of registering with the study, participants were asked to tick: Sex: ‘Female’ or ‘Male’. All participants that gave data for decision acuity and imaging analyses ticked one or the other box. It was not clarified if some understood the question as ‘gender identity’, socially attributed or biological category. Due to the phrasing ‘Sex:’ we expect that most participants understood the question to mean ‘self-reported estimate of biological sex’, but this is a tentative interpretation.

In addition, they were screened for absence of a history or presence of mental health disorder, neurological or major health problem, or learning disability. Initial screening was by self-report but was confirmed by SCID-II interview and IQ testing.

We supplemented this non-healthcare-seeking sample with 50 young people recently diagnosed with DSM-5 major depressive disorder. Of these, 38 gave decision-making battery data for decision-acuity analyses (M = 11,F = 27). Thus, the main sample was representative of the healthy wider population, but a smaller depression group was also analyzed to test whether the structure of decision-making and the relevant brain measures identified in the healthy population also extended to this health-seeking group. The depressed cohort was excluded from MRI analyses reported here.

Participants (and their parents, if less than 16 years old) gave informed consent to participate in the study. The study was approved by the Cambridge Ethics Committee (12/EE/0250).

### Method details

#### Sample size estimation

Our key sample size estimation pertained to the neuroimaging sample, and resulted in the estimate of N = 300. The cognitive-task sample was then as large as study resources allowed, including resources needed to re-telephone participants who had initially given consent but did not immediately respond to an invitation of follow-up, up to achieving a follow-up rate of at least 70%. In summary, estimation of the key, neuroimaging cohort sample size proceeded as follows.

At the time of study design, there were no specific studies to provide a rigorous analysis for rsFC developmental, longitudinal sample size estimation. We therefore relied on a roughly comparable study which allowed for imaging developmental effect. This study used a cohort of 387 participants, who provided 829 structural MRI scans (Giedd, 2004). We thus aimed for 300 participants, a number which was logistically accessible, and optimized power by selecting parameters (age minimum and width) of age-bins and follow-up intervals, using published gray-matter volume data as a proxy for the individual variation that we should have power to detect. Quadratic growth curves were fitted to the data from the published study above, and study parameters varied *in silico* to minimize variance of the estimated parameters of the growth-curves. Simulations showed a plateauing of efficiency if the overall age range was reduced to less than 10 years, or the width of age-bins to less than 2 years. Parameter accuracy improved with follow-up interval and deteriorated if the follow-up was shorter than 6 months. Therefore, we aimed for 5 age bins times 2 years width, and selected a minimum interval of 12 months, aiming at about 18 month average. This was well above 6 months, reduced the chance of demographic loss (moving far away) and allowed adequate time to repeat invitations for participants that did not immediately respond to follow-up invitations.

#### Decision-making Task Battery

We selected seven tasks tapping fundamental decision-making with evidence linking them to both mental health symptoms and neural mechanisms (Table 1 in main text). First, a *Go-NoGo task* (Guitart-Masip et al., 2012) provided measures relevant to sensitivity to rewards and Pavlovian bias. Second, an *approach-avoidance task* measured the balance of seeking rewards versus avoiding losses (Bach et al., 2014). This is likely to be relevant to everyday risk-taking by young people. Third, a *risk preference task* (Symmonds et al., 2011) complemented this, focusing on widely accepted economic measures of risk-taking (Bach et al., 2020; Rigoli et al., 2016). Fourth, we assessed *inter-temporal discounting*, learning about the preferences of others and finally peer influence (Moutoussis et al., 2016; Nicolle et al., 2012). Discounting has been shown to be important in a range of psychiatric disorders (Bickel et al., 2012) and so are issues of thinking about others (Sripada et al., 2009) and peer influence (Kerr et al., 2012). Fifth, we included an *information gathering task* (Moutoussis et al., 2011) as this has been consistently shown to be relevant to psychotic symptoms (Lincoln et al., 2010b) as well as the fundamentals of decision-making (Dayan, 2014). Sixth, a *Trust Task* was used as a measure of complex social cognition especially relevant to disorders of interpersonal function (Fett et al., 2012; King-Casas et al., 2008). Seventh, *a two step task* assessed the role of habitual versus planful mechanisms in decision-making (Daw et al., 2011). The battery was implemented using MATLAB (MATLAB, 2012) using the Cogent toolbox (see acknowledgments). Trained research assistants directed the participants through the battery.

In terms of remuneration, participants received a flat fee but were also (truthfully) told that they would be paid extra according to their earnings in the tasks. They were informed that there would be a substantial amount of luck in each task, but those who completed the tasks carefully would expect to earn about 2.5 pounds extra per task. Participants did not see earnings for each trial, because tasks differed greatly in their delivery and we did not want to display varying amounts of money to avoid additional Pavlovian motivational effects. Instead, participants were told that ‘roughly, each good decision in each task is worth approximately the same’, a statement which provided a reasonable reflection of the true state of affairs. The sole element of deception in the battery was that during the interpersonal tasks participants were told that their play partner was a peer, whereas in reality it was a computer agent. However these agents were simulating as closely as possible the performance of healthy people who had the same demographics as the participants. Participants were debriefed at the end of all testing.

Earnings were added to their compensation for the day’s testing, except for the Interpersonal-Discounting task. Here, participants were paid at one of their chosen delays, randomly chosen from all the trials in the task, if they chose a larger but delayed payment. This was paid in Amazon vouchers.

The order of the tasks was subject to constrained randomization. We first piloted the battery in 15 participants, of whom we asked detailed feedback as to how interesting and how tiring they found each task, as well as free-form comments. On the basis of this we avoided putting the more tiring or less interesting tasks near the end of the battery, in order to minimize the effect of fatigue. This resulted in eight different task sequences, one of which was given at random to participants. After the first 40 participants were recruited we performed an interim analysis to compare performance in this battery of shortened tasks as compared to the full-length versions. Performance in each task showed followed the pattern of performance in the original, except the Two-Step task. Here participants as a group showed only just-detectable goal-directed decision-making. As this would greatly reduce the task’s usefulness we improved the pre-task training and instructions and discarded this first ∼10% of data for this task, with satisfactory results.

Tasks lasted 8-30 min each, giving an overall duration of 2 ¾ – 2 ¾ h, including one obligatory break and as many extra between-task breaks as the participant asked for. Good performance attracted proportionally greater fees in real money.

Key measures were first extracted from each task according to published methodologies. These key measures assess fundamental aspects of decision-making, namely sensitivity to rewards and losses, attitudes to risk, inter-temporal and reflection impulsivity, pro-sociality and model-basedness. 820 participants (including all scanned participants) yielded usable data across tasks. The approach-avoidance task, the information gathering task, and the trust task required some adaptations that are listed below.

We were interested in whether common factors operated across domains of decision-making. We therefore pre-processed the data to reduce strong correlations among measures within-task, which would otherwise dominate the factor analysis, as is described in the supplemental information. In total we formed 32 measures, listed in Tables 1 and S1.

The *approach avoidance task* was originally described in Bach et al. (2014) was adapted for the purposes of this study. Because of time constraints we reduced the number of threat contexts from three to two, which we call two ‘predators’ corresponding to low and high threat. Also, different from the previous study, epoch duration did not depend on threat level. That is, an epoch ended after a random duration, independent of whether the predator woke up or not. Finally, the number of epochs was reduced to 1/3 of the original, so that the task took about 23 min to complete.

Based on the previous work (Bach et al., 2014), we collected a large number of behavioral descriptive measures and performed an exploratory factor analysis of these (substantially correlated) measures. We found that the first three factors could be meaningfully interpreted in decision-making terms, namely as sensitivity to the level of threat in the environment (‘threat sensitivity’), sensitivity to features increasing probability of loss within an environment (‘loss sensitivity’) and measures of overall performance (‘performance’). As might be expected, this third ‘performance’ factor loaded more highly in *d* (Figure 1) but still did not exceed the threshold of 0.25 that we used for inclusion in confirmatory analyses (below).

The ‘cover story’ and graphics of the *Information Gathering task* were adapted from the work of Lincoln and coworkers (Lincoln et al., 2010a, 2010b). On the basis of previous work (Moutoussis et al., 2011) we reduced the maximum number of samples of information per trial in order to increase the impact of the approaching end (urgency). We first presented participants with an uncosted-information gathering version of the task for 10 trials. This had deliberately non-specific instructions to maximize the chance that participants would bring their own, subjective cost structure to bear and because, somewhat unexpectedly, such uncosted, scarce-instruction versions of the task has produced some of the most consistent results in clinical and subclinical samples. We then presented them with 10 trials with more specific instructions. Participants started with 100 points and had to pay 10 points for each item of information they requested. We employed a maximum-likelihood fit of the bayesian-observer model from Moutoussis et al. (2011).

In order to analyze the *Trust task* we adapted the measures described by Fett et al. (2012). Following these researchers, we considered whether participants increased or decreased their offer at each move in response to observing their partner increase or decrease theirs. However we considered the fractional change in contribution, i.e., the change in the fraction of play-money that could have been given. This entails the hypothesis that each player considers the other as ‘messaging’ them from a baseline of their financial means, not in absolute terms. We then considered the vector in the 2-dimensional space of (fractional-change-of-Investor by fractional-change-of-Trustee) formed for each round of play. We classified this in the same way as Fett et al. (retaliating, repairing, honoring, disrupting) as the angle between the vector and the change-of-Investor axis increased from −180 to 180 degrees. Again using the (rather crude) approximation that strategy remains the same throughout the 10 rounds of the game, we added the vectors for each of the rounds to determine the character of the game as a whole. The orientation of the resultant vector characterizes the whole exchange – both Investor (our participant) and Trustee (the computer). As all investors played the same computer program, this vector can be seen as the type of exchange that the participant elicited.

In the event, orientations showed a clear bimodal distribution, either around zero degrees (an exchange based on coaxing the Trustee) or around −3π/4. The latter represents an exchange where each party is responding to the other’s reduction in contribution with their own reduction. We might speculate that participants attempt to signal ‘if you won’t be generous, I won’t either’. The two-cluster distribution could in turn be fitted reasonably well with a single straight line spanning retaliatory to coaxing exchanges. The ‘trust building’ index in Tables 1 and 2 corresponds to the participant’s position along this line.

#### MRI data acquisition

MRI scans were acquired on three identical 3T whole-body MRI systems (Magnetom TIM Trio; VB17 software version; Siemens Healthcare): two located in Cambridge and one located in London. Reliability of the MRI procedures across sites has been demonstrated elsewhere (Weiskopf et al., 2013). Structural MRI scans were acquired using a multi-echo acquisition protocol with six equidistant echo times between 2.2 and 14.7 ms, and averaged to form a single image of increased signal-to-noise ratio (SNR); TR = 18.70 ms, 1.0 mm isotropic voxel size, field of view (FOV) = 256 × 256, and 176 sagittal slices with parallel imaging using GRAPPA factor 2 in anterior-posterior phase-encoding direction. Resting-state blood-oxygen-level dependent (BOLD) fMRI (rsfMRI) data were acquired using multi-echo acquisition protocol with three echo times (TE = 13, 31, 48 ms), TR of 2420 ms, 263 volumes, 3.8 mm isotropic voxel size, 34 oblique slices with sequential acquisition and a 10% gap, FOV = 240 × 240 mm and matrix size = 64 × 64 × 34. The duration of the functional scan was approximately 11 min.

#### Connectivity Analysis

The rsfMRI data were denoised with multi-echo independent component analysis (ME-ICA) (Kundu et al., 2017). ME-ICA leverages the echo time dependence of the BOLD signal to separate BOLD-related from artifactual signal sources, like head motion. The functional images were normalized to MNI space by composing a rigid transformation of the average functional image to the participant’s structural image and a non-linear transformation of the structural image to the MNI template, and finally smoothed with a 5 mm full-width-at-half-maximum Gaussian kernel. Following Smith et al. (2015), group-ICA was applied to the pre-processed fMRI baseline data to decompose it in 200 nodes, 32 of which were identified as artifacts by visual inspection and excluded. The remaining 168 nodes are either confined brain regions or networks formed by regions where BOLD signal time-series are strongly correlated. Multiple spatial regressions against the group-ICA spatial maps were used to estimate time-series for each network and subject, for both baseline and follow-up scans. RsFC matrices (168 × 168 nodes) were then computed using partial correlation with limited L2 regularisation (Smith et al., 2011). All these preprocessing steps were conducted with the ME-ICA toolbox (https://afni.nimh.nih.gov/pub/dist/src/pkundu/README.meica) and the FMRIB Software Library (FSL, https://fsl.fmrib.ox.ac.uk/fsl/fslwiki/). As shown in Figure S4 and recent studies of FC reliability (Noble et al., 2017), overall reliability of individual functional connections is low, though some connections display moderate to high reliability. We thus used multivariate methods combining multiple FC values as a strategy to compensate for the low FC of individual connections.

The obtained rsFC values were used as features in a sparse partial least-squares (SPLS) model to predict two outcome measures of interest (decision acuity and IQ composite scores). SPLS (Chun and Keleş, 2010; ‘spls’ R library, https://cran.r-project.org/web/packages/spls/) is a multivariate regression model that simultaneously achieves data reduction and feature selection. It has application in datasets with highly correlated features and sample size much smaller than the total number of features, as was the case in the present study. SPLS models are governed by two parameters (number of latent components and a threshold controlling model sparsity) that were adjusted using a nested cross-validation scheme (i.e., using data in the training dataset only) with 10-folds (Figure 2).

Predicted scores were estimated by 20-fold cross-validation repeated 5 times. For each training-testing partition we performed the following steps. To elucidate whether the predictions were driven by rsFC values independently of age, sex or covariates of no interest (see below), we fitted a linear model to the training dataset and regressed out from the target variable (in both training and testing datasets) age, sex and their interaction as well as brain volume, scanning site and head-motion-related parameters. Head motion is known to originate spurious correlations that bias connectivity estimates and therefore (besides the ME-ICA preprocessing explained above) we regressed out average framewise displacement (FD), a summary index of the amount of in-scanner motion (Power et al., 2012), and the degrees of freedom resulting from the ME-ICA denoising, which may differ across subjects depending on how much nuisance variance is removed from their data. As an additional control for head motion, subjects whose mean FD was above 0.3 mm were not included in the analysis. We also standardized both training and testing data with respect to the mean and standard deviation of the training data (separately for each feature). As a first step to filter out uninformative features and speed up computations, only those significantly (p < 0.05) correlated with the outcome variable in the training dataset were entered in the SPLS model. We then used a bagging strategy where data were resampled with replacement 200 times and as many SPLS models were fitted to the resampled datasets, and their feature weights averaged to produce a final model. The purpose of this step was 1) to improve the generalizability of the final average model and 2) to allow estimation of the stability of the feature weights selected. The final, average model was used to compute the predicted scores for the testing partition. The same procedure was repeated for all folds to obtain one predicted score for each subject, where the predicted score for each participant depended only on data from other subjects in the sample. These procedures were implemented with R (https://www.r-project.org/) and MATLAB (https://www.mathworks.com).

#### Network node community structure

To enhance our understanding of the anatomical distribution of the predictive connections, we performed a ‘virtual lesion’ analysis (Dubois, et al., 2018), which entails assessing the performance of the model when it is trained only on subsets of connections instead of the full ensemble. First, we partitioned the set of nodes into disjoint modules or communities (to some extent analogous to large-scale functional networks; Smith et al., 2009) formed by nodes which displayed high connectivity among them but lower connectivity with nodes in other modules. We obtained the community structure directly from our dataset instead of relying on previous partitions that have been derived from adult connectomes (Ito et al., 2017; Power et al., 2011), because brain connectivity of adolescents and adults is known to differ (Fair et al., 2009).

To produce the partition, we averaged the baseline rsFC matrices across participants and removed negative entries. The resulting matrix was submitted to the Louvain community detection algorithm for weighted graphs (Blondel et al., 2008) and this partition was refined using a modularity fine-tuning algorithm (Sun et al., 2009). Since the algorithm is not deterministic, it was applied 100 times and the results gathered in a nodes x nodes consensus matrix that indicates the frequency by which the corresponding node pair was assigned to the same module. The consensus matrix was partitioned repeatedly until convergence. The algorithm depends on a parameter γ that controls the resolution (which determines the ensuing number of modules). We adjusted this parameter to maximize the normalized mutual information between solutions at different resolutions. The optimal value of γ ensures the most stable partitioning and in our dataset (γ = 2.7) led to a solution with 14 modules, a number that yielded interpretable modules and is on par with the cardinality used in previous studies. These analyses are similar to those reported in (Geerligs et al., 2015) and were performed with the Brain Connectivity Toolbox (Rubinov and Sporns, 2010; http://www.brain-connectivity-toolbox.net) for MATLAB. Having parcellated the connectome in the 14 modules, we trained the prediction model for each one of them using only connections implicating nodes in that module (i. e. either connections among nodes in the module or connections between nodes in the module and the rest of the brain). We employed the same module decomposition in the analysis concerning the follow-up dataset.

### Quantification and statistical analysis

#### Derivation, validation and psychometric correlates of Decision Acuity

We tailored analysis to test the hypothesis that a few (around three) dimensions of covariation would meaningfully load across decision-making measures, expecting reward sensitivity, risk preferences, goal-directedness and prosociality to be represented in these dimensions. We allowed, however, the data to determine the number of factors in the model. We used an exploratory-confirmatory approach to establish the structure of the factor model using the baseline data. Then, we made use of the longitudinal nature of our sample to test the temporal stability and predictive validity of the key derived measure.

Task measures at baseline only were first transformed to near-normal marginal distributions using logarithmic or power-law transforms, imputed for the small percentage of missing values using the R package ‘missMDA’, then randomly divided into a ‘discovery’ and ‘testing’ samples. N = 416 participants were used for exploratory common factor analysis (ECFA) and 414 were used for out-of-sample testing. We found loadings on the first ECFA factor, likely to be most important, to vary smoothly across all parameters, and the great majority of loadings to be lower than the conventional threshold of 0.4 used to construct structural equation models for confirmatory FA (Muthén and Muthén, 2008). Items had high uniqueness, as expected. These results were much like the final total-sample FA illustrated in Figure 1. Therefore, rather than claim that certain decision parameters were important and others were not in providing a measure of the underlying latent variable, we allowed for all decision-making items to contribute, recognizing that individual item weights would be poorly estimated, but expecting that the resulting overall scores would be well estimated. We tested this by comparing (i) discovery versus test samples and (ii) purposeful half-splits of the population with respect to sex and age (see supplemental information, section B). The exploratory analysis furthermore suggested that our objective need not be to determine a ground-truth number of factors, as higher order factors were dominated by single tasks and hence were of no interest here. Our criterion for including higher-order factors then was whether higher dimensional models were likely to result in better score estimates for the low-order factors, which were of interest.

The maximum number of factors for the exploratory-confirmatory analysis was 8, estimated by parallel analysis (Figure S1). The R library ‘nFactors’, and specifically R functions ‘eigen’,’parallel’ and ‘nScree’ were used to estimate the scree-plot based number of exploratory factors to retain illustrated in Figure S1. Based on these, models from 8 down to 1 factors were derived with function ‘fa’, using ordinary least-squares to find minimum-residual (minres) solutions. The models estimated from the ECFA were then tested on the confirmation dataset using structural equation modeling in R (Fox, 2006). Criteria of under-determination, Bayesian Information Criterion (BIC), and comparative fit index (CFI) were used to compare including an increasing number of factors. In the confirmatory factor analysis, a threshold of 0.25 was adopted as very few loadings on the first factor exceeded the conventional threshold of 0.4 (See Figure 1 in the main text). Considering the test set of 414 participants only, we found that model fit as indexed by the BIC and CFI improved from 1 to 4 factors. However, 5 factor and more complex model fits did not converge on the test set. According to these criteria we considered a model of four factors to be most parsimonious and robust, but we also considered the stability of the latent constructs derived to make a final choice of factor-analytic model. Within the range of three to five factors, *d* scores were not sensitive to the exact number of factors, scores being correlated with r > 0.9, p ∼0, with the score obtained from the 4-factor solution. We thus opted for a 4-factor model for all subsequent analyses.

We then tested whether decision acuity as a construct was stable with respect to (i) the random discovery/confirmation split (ii) median-split age and (iii) sex using the baseline data. We examined how closely scores for a certain subgroup (below median age for (ii), ‘Male’ for (iii)) based on ECFA of the group itself agreed with scores for the same individuals based on FA weights derived from the opposite (i.e., above median age or ‘Female’) group. We examined the construct stability of decision acuity by correlating component *d* scores on half the sample with the same scores derived from the first ECFA component on the other half of the sample. Here we were not primarily interested in the factor structure of decision-making, but in the stability of the construct of decision acuity. We thus divided the sample into two subgroups either by age (at 19) or by sex. We argued that if the construct itself was stable across age (and sex), then the decision acuity factor score for each participant could be calculated either using the factor loadings derived from the participant’s own group or indeed the opposite one. Individuals with substantially differing scores would indicate that a different latent construct organized decision-making across the subgroups. If, for example, *d* was an invariant latent construct with respect to age, then the pattern of loadings derived from older participants would give the same scores when applied to younger participants as and ECFA on the young participant data themselves (Figure S3). *d* was highly stable across the discovery-confirmation random split (0.99 confidence interval for r(exploratory based on confirmatory loadings, own exploratory) = 0.976,0.985), as well as age CIr(young|old, own young) = 0.969,0.9811). Its stability across gender was satisfactory but significantly lower, evidencing a small degree of sexual dimorphism CIr(male|female, own male) = 0.820,0.887. Fit indicators were similar for the whole sample and for each split (e.g., RMSEA 90% CIs for females, males, younger, older and all were 0.051-0.061, 0.052-0.062, 0.054-0.064, 0.046-0.056 and 0.052-0.058 respectively). None of the analyses was materially affected by excluding from the sample of 830 participants the 50 who had a diagnosis of DSM5 depression.

Finally, we tested for external validity of decision acuity in correlating with (iv) mental health scores for symptomatology and dispositions, using bifactor scores and (v) patterns of functional brain connectivity, as described in Results.

The follow-up battery did not contain one of the baseline tasks, and had minor differences (but the same derived parameters) for two further tasks. In order to perform longitudinal analyses, we adopted a conservative approach, estimating a measure of decision acuity based on the final stage of the baseline analysis, but retaining only the weights for the six tasks that were assessed longitudinally. We checked that this more approximate measure adequately captured individual variability of the baseline sample, which was the case (r = 0.98, p undetectable) and therefore used in the longitudinal analysis baseline scores derived from these six tasks. We then derived the follow-up decision acuity estimates as follows. We first applied the same approximate-gaussianization transforms to each follow-up measure. Next, we z-scored each follow-up measure using the mean and standard deviation of the respective (transformed) baseline measure. Finally, we applied the weights for these 6 tasks derived from the baseline factor analysis. Thus, we took the follow-up measures of decision acuity to have exactly the same structure as baseline, so that it could be used to compare absolute changes in this measure. Finally, in analyses correlating follow-up symptoms with decision acuity, and as decision acuity and IQ were measured typically six months after symptoms and hypothesized to be trait-like, we interpolated follow-up decision acuity and IQ measures to the time of symptom measurement.

For the longitudinal analysis, we used a linear mixed effects approach. As the structure of decision Acuity was fixed by the procedure above, we did not split the follow-up sample into test and discovery sets. We used Bayesian Information Criterion to select the statistical models by which we tested for inter-relations between decision acuity and key psychometric variables. For all the following analyses, N = 571 for the follow up sample. Developmental time in this accelerated longitudinal design is represented both by age-at-recruitment, and by the time interval between test waves. Both recruitment procedures and development itself may mean that these two measures of developmental age may in practice affect our dependent variables differently. We therefore first checked if LME modeling over baseline and follow-up with age as a random effect, in addition to a random intercept for each participant, improved model fit. In fact, it worsened model fit (BIC = 5974.5; logLik = −2965.512; versus BIC = 5960.0, logLik = −2965.529), so we did not include age as random effect in further analyses. In further analyses involving IQ, we used the raw matrix and vocabulary WASI IQ subscores and modeled age explicitly, rather than use standardized IQ subscores. This is because we noticed that the standardized WASI total IQ in our sample was associated with age (r Pearson = 0.135, p = 0.00011, *r*^2^ = 0.017) at baseline. This indicates that our sample had a different age dependence of IQ scores than the reference one (Axelrod, 2002). Therefore, we regressed *d* for raw IQ subscores while covarying for age, in effect accounting for variation in IQ ability independent of whether this was due to age or self-selection.

We also formed a performance measure across tasks, in order to check the interpretation that *d* reflects better decision-making. First, we excluded the discounting and Roulette tasks, as these specifically probed the balance of amounts won versus other dimensions of the return, namely its delay and uncertainty respectively. Second, we excluded the Approach-Avoidance conflict task, as one of the measures by which it entered the estimation of *d* was judged to be too close to a performance measure already (the third common-factor scores of the within-task factor analysis; see above). Parenthetically, this measure loaded modestly in the expected direction onto *d*, i.e., positively, with a weight of 0.24 and high uniqueness (Figure 1). We then t-scored winnings within each of the Go-NoGo, Information Gathering, Investor-Trustee and Two-step tasks, and averaged these scores across tasks. The Pearson raw and partial correlation table between this task-performance measure, *d* and WASI total IQ, shown in Table S2, supported the interpretation of *d* as conducive to profitable decision-making above and beyond IQ.

#### Predictive performance of Connectivity Analysis

We assessed predictive performance as the Pearson correlation coefficient *r* between measured *d* and (cross-validated) predicted *d* (*d*_*pr*_), averaged across repetitions of the cross-validation splits. After Fisher transformation, the null distribution of *r* should follow a zero-centered Gaussian distribution. In order to appraise significance, we estimated the variance of this distribution by generating 100 random permutations of the target variable (Winkler et al., 2016) and repeating the model-fitting procedures mentioned above, separately for each fold. We then derived p values for the observed *r* from the estimated null distribution. We assessed predictive performance for a model based on the full set of connections, as well as for models trained on the subsets of connections corresponding to the modules described in the previous subsection.

To demonstrate that the relationships between connectivity and decision acuity were stable over time and replicate, we used the model estimated at baseline to predict *d* based on the follow-up rsFC data for modules that were significant at baseline. Given that the data at baseline and follow-up are not independent, we kept the same cross-validation fold structure in both datasets, so that the prediction of a subject at follow-up did not involve their own rsFC baseline data, as this would have inflated the estimates of predictive performance at follow-up.

#### Connectivity patterns predictive of *d* versus IQ

For imaging analyses, we derived a composite score of IQ by averaging standardized vocabulary and matrix IQ subscores, rather than using the standardized WASI score, because of two reasons. First, we wanted analyses involving both age and IQ to have a straightforward interpretation where IQ represents a measure of raw ability, as opposed to age-standardized ability, and explicitly test for age-dependence separately. Second, we found evidence (Results) that our sample was different from the original on which standardized scores were derived, and hence the standardization procedure might be invalid. Next, we trained models both on the complete set of connections and the subsets corresponding to the individual modules to predict the IQ composite scores, as we had done previously to predict *d*, yielding IQ_*pr*_, and assessed predictive performance for each of the modules separately. To compare the connectivity patterns that were predictive of *d* with those predictive of IQ, for each of the modules we assessed the partial correlation between *d* and *d*_*pr*_ when controlling for *IQ*, and the partial correlation between *IQ* and *IQ*_*pr*_ when controlling for *d*. In all these analyses we corrected for age, sex and imaging-related confounds as above.

## Supplemental information

Document S1. Figures S1–S4, Tables S1–S3, and The Neuroscience in Psychiatry Consortium member list

Document S2. Article plus supplemental information

## Footnotes

Supplemental information can be found online at https://doi.org/10.1016/j.neuron.2021.04.019.
