# Supplementary material for: Decision-making ability, psychopathology, and brain connectivity
Source: Neuron. 2021 Jun 16;109(12):2025–2040.e7. doi: 10.1016/j.neuron.2021.04.019 (PMC8221811; doi:10.1016/j.neuron.2021.04.019)
Supplement: Document S1. Figures S1–S4, Tables S1–S3, and The Neuroscience in Psychiatry Consortium member list [file mmc1.pdf]

Neuron, Volume *109*

Supplemental information

Decision-making ability,

psychopathology, and brain connectivity

Michael Moutoussis, Benjamín Garzón, Sharon Neufeld, Dominik R. Bach, Francesco Rigoli, Ian Goodyer, Edward Bullmore, NSPN Consortium, Marc Guitart-Masip, and Raymond J. Dolan

A. Decision-making battery measures

Learning rate (D)	**Learning rate, Two -step task (transformed)**
Perseveration (D)	**Perseveration parameter, Two-step task**
Inverse Temp. (A)	**Inverse temperature, Go-NoGo task (transformed)**
Inverse Temp. (D)	**Inverse temperature, Two -step task (transformed)**
Initial Investment (F)	**Initial investment in partner, Investor-Trustee task**
Aversive lrn. Rate (A)	**Aversive learning rate, Go-NoGo task**
Appet. lrn. rate (A)	Appetitive learning rate, Go-NoGo task
Appr.-Avoid fact.3 (C)	'Performance factor', Approach-Avoidance task
Cooperativeness (F)	Degree of cooperative responding, Investor-Trustee task
Appr.-Avoid fact.1 (C)	'Sensitivity to overall threat level', Approach-Avoidance task
Appr.-Avoid fact.2 (C)	'Sensitivity to increasing hazard', Approach-Avoidance task
Model-basedness (D)	Model-basedness, Two-step task (transformed)
No ext. price cost (E)	Subjective cost of samples, uncosted Info. Gathering (transf.)
Eligibility (D)	Eligibility trace parameter, Two -step task
Ext. priced cost (E)	Subjective cost of samples, costed Info. Gathering (transf.)
Action bias (A)	Bias towards action, Go-NoGo task (transformed)
Skew preference (B)	Sensitivity to outcome skewness, Econ. preference task
Epistemic trust (F)	Epistemic trust parameter, delegated discounting (transf.)
Gambling preference (B)	Overall preference for gambling, Econ. preference task
Temp. Other-choice (F)	Variability of choices-for-other, delegated discounting (transf.)
Pavlovian RT diff. (A)	React. time diff. between conditions, Go-NoGo task (transf.)
Risk preference (B)	Risk aversion, Econ. preference task
Lapse rate (A)	Lapse rate, Go-NoGo task (transformed)
Reactiveness (F)	Reactiveness to other's offers, Investor-Trustee task
Pavlovian bias (A)	Pavlovian bias, Go-NoGo task (transformed)
Taste uncertainty (F)	**Taste uncertainty, delegated discounting (transf.)**
Temp.- ext. priced (E)	**Decision temperature, costed Info. Gathering (transf.)**
Lapse rate (F)	**Lapse rate, delegated discounting (transformed)**
Temporal discounting (F)	**Temporal discounting, delegated discounting (transf.)**
Average RT (A)	**mean log-Reaction Time, Go-NoGo task**
Temp. - no ext. price (E)	**Decision temperature, uncosted Info. Gathering (transf.)**
Exp. Value sensitivity (B)	**Sensitivity to expected value of outcome, Econ. preference task**

Table S1, related to Figure 1. Key to the labels of cognitive measures, in the order of loading onto decision acuity. Green - load positively; Blue - load negatively; Bold - exceed 0.25 in loading. Letters in brackets refer to Table 1 in main text.

B. Factor analysis and validation of Decision Acuity

B1. Exploratory - Confirmatory analyses

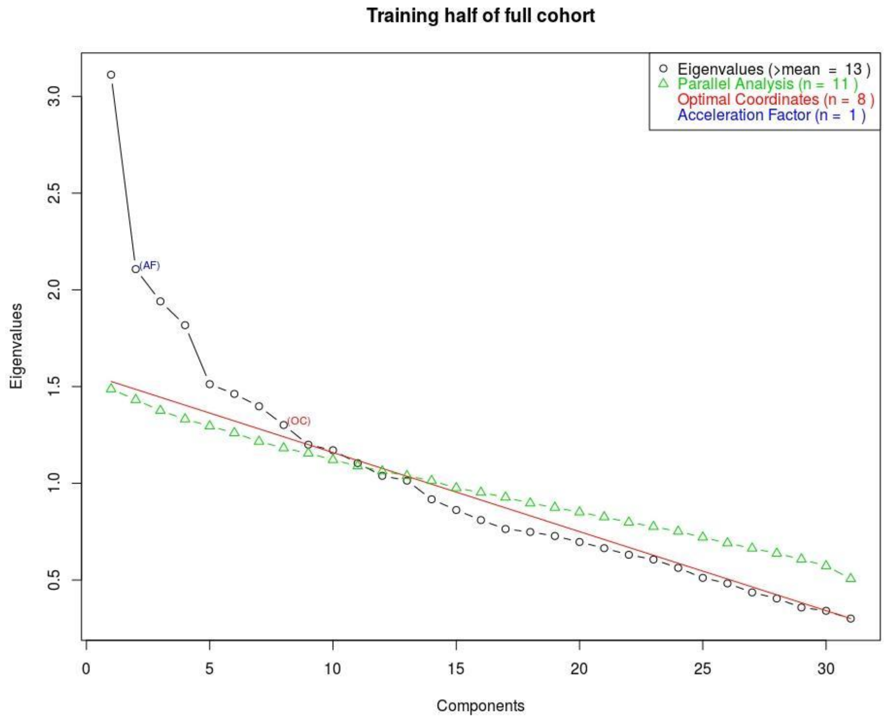

Figure S1, Related to STAR Methods. Parallel analysis to determine optimal number of factor-analytic components for the 'Discovery dataset', N = 416, and 32 variables.

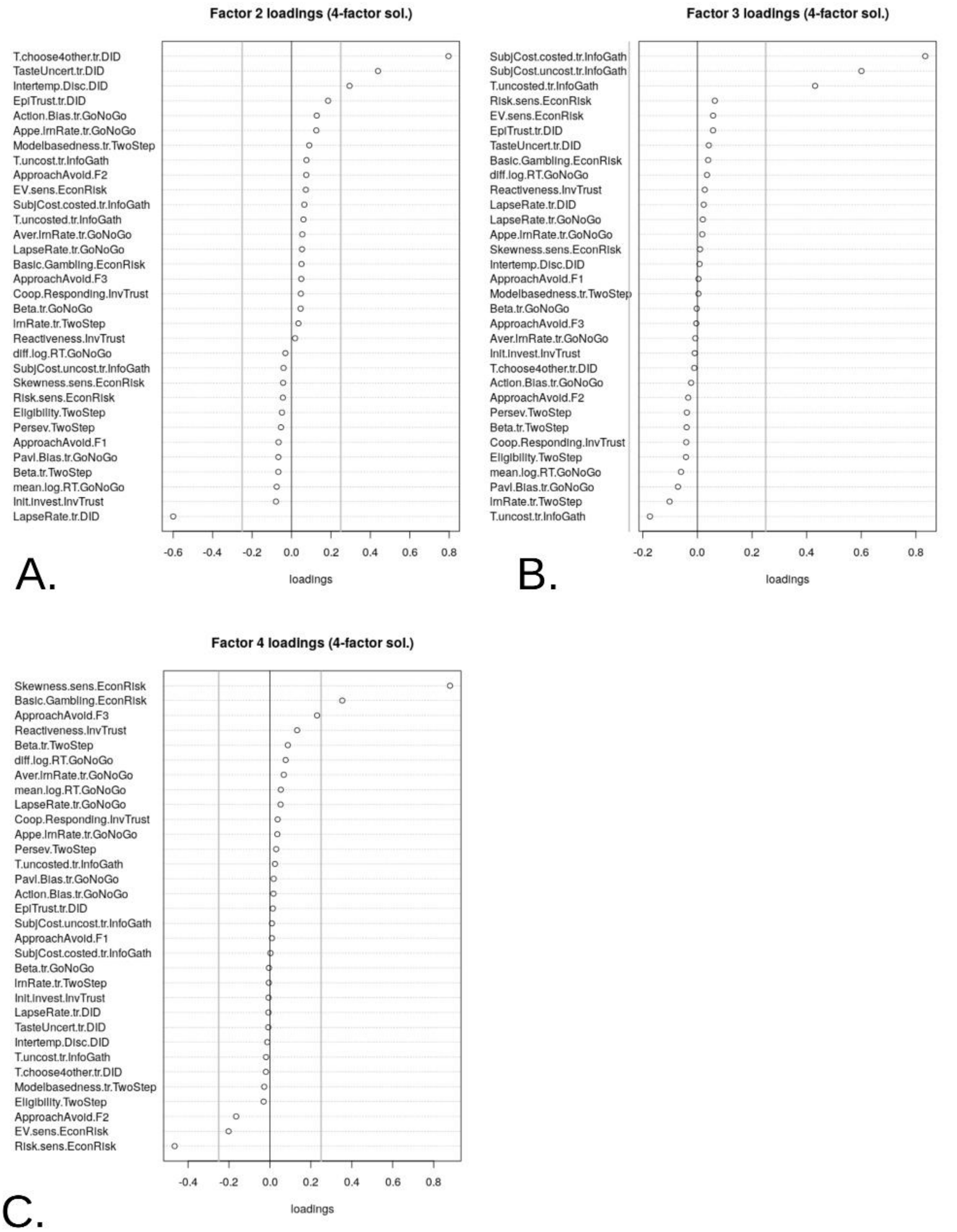

Figure S2, Related to STAR Methods. Factor loadings for factors 2 to 4, exploratory common factor analysis on the whole sample. loadings with absolute value over the noise floor or over 0.25 (gray lines) are exclusively from: **A.** Delegated Discounting task for Factor 2 **B.** Information Gathering task for Factor 3 and **C.** Economic risk preference task for Factor 4.

B2. Stability Analysis

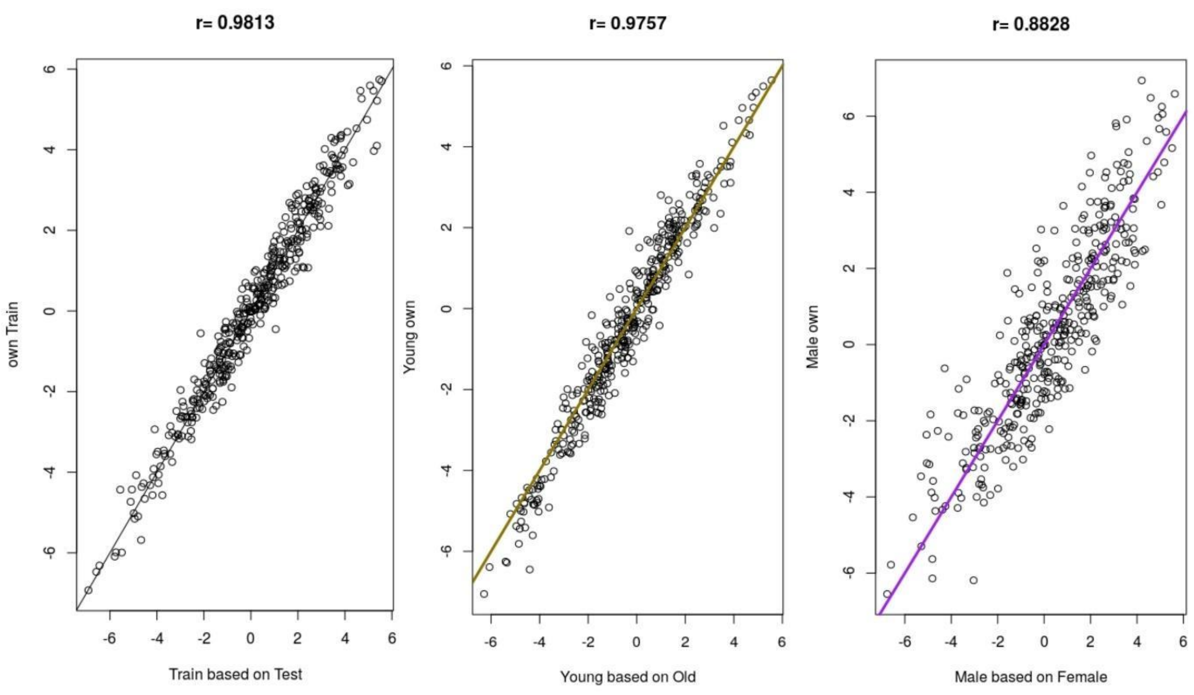

A. B. C.

Figure S3, Related to STAR Methods. Stability of the construct of decision acuity with respect to random variation in the data, age or sex. In each case, component factor scores for half the sample based on ECFA of that same half-sample is predicted by component scores for the same individuals, but based on the construct (i.e., factor loadings) derived from the opposite half of the data. **A.** Exploratory-confirmatory split gives a very high correlation (r=0.98, p ≅ 0.0) attesting to the reliability of the construct **B.** Median split at age= 18.54 years. Very high correlation (r=0.98, p ≅ 0.0) attests to the stability of the construct in young adults vs. teenagers. **C.** Female-male split shows somewhat lower correlation (r=0.88, p ≅ 0.0), suggesting that the same 'average' construct can be used in both sexes, but also that subtle sexual dimorphism exists.

C. Additional associations of d with performance, symptoms and IQ

C1. *d*, performance and IQ

		d	WASI IQ
task performance	partial	r=0.42, p <1e-10	r=0.05, p=0.14
	raw	r=0.50, p <1e-10	r=0.30, p<1e-10
d	partial	-	r=0.44, p<1e-10
	raw	-	r=0.51, p<1e-10

Table S2, Related to Table 2. Relations of d and IQ with overall performance in four key tasks.

C2. Relations of total IQ and decision acuity with symptom and disposition factors

Psych. factor	*r* psy-IQ	*p* psy-IQ	*r* psy-d	*p* psy-d
p-factor	-0.06566	0.06878	-0.10516	0.00334
self-confidence	-0.05557	0.12362	-0.08575	0.01681
misbehaviour	-0.08504	0.01835	**-0.152**	**2.0E-05**
worry	0.07159	0.04718	**0.13439**	**0.00017**
aberrant thinking	**-0.15563**	**1E-05**	**-0.16718**	**< 1E-05**
mood	-0.01269	0.72524	0.01864	0.60389
Sociality	-0.07832	0.02599	**-0.13253**	**0.00014**
social sensitivity	0.06021	0.08721	0.01161	0.74023
sensation seeking	0.03853	0.27396	0.01699	0.62744
effortful control	0.08922	0.01117	0.05352	0.12611
suspiciousness	-0.10465	0.0029	0.01312	0.70782

Table S3, Related to Table 2. Raw correlations of total IQ and decision acuity with symptom and disposition factors at baseline. The Bonferroni-corrected for 22 comparisons correlations at $p<0.05$ are shown in bold. IQ mostly correlates with 'aberrant thinking' symptoms (schizotypal / obsessional) while decision acuity additionally relates to 'misbehaviour', 'worry' and most importantly the dispositional general factor 'Sociality'.

D. Brain connectivity analyses

D1. Reliability of functional connectivity

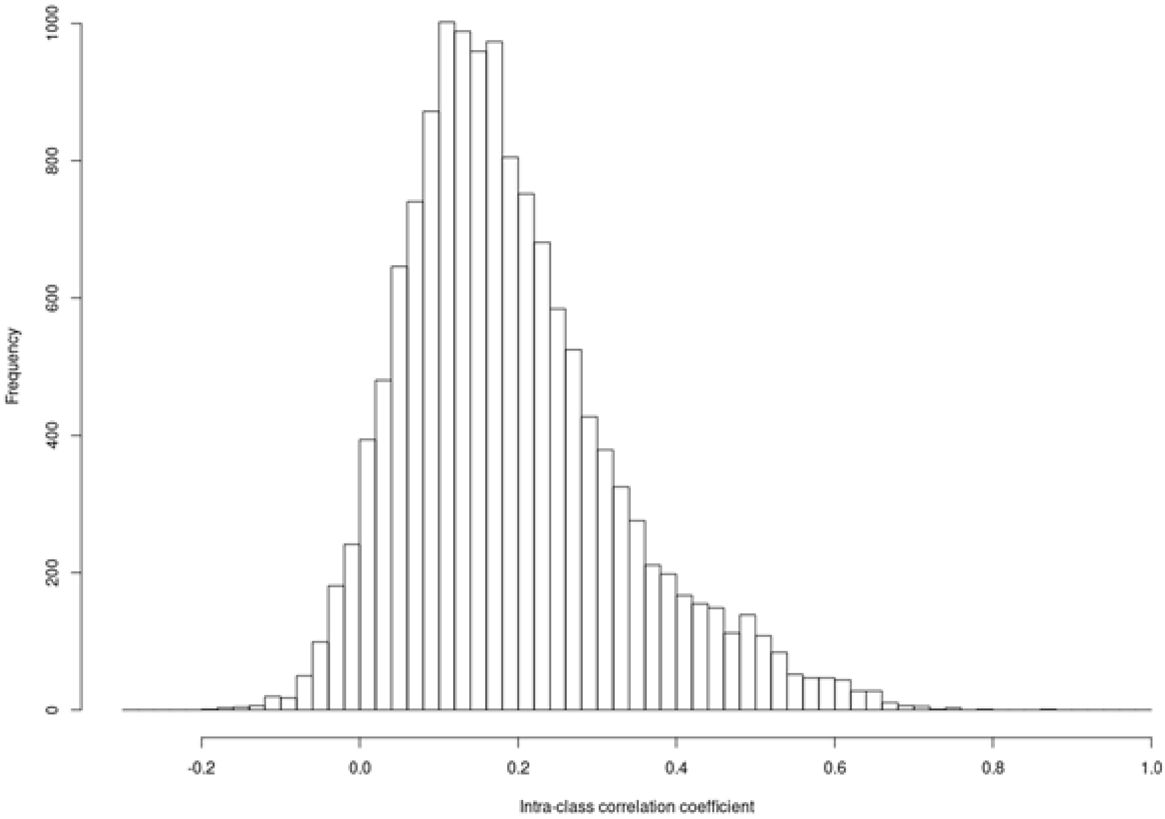

Figure S4, Connected to STAR Methods and Figures 3-5. Histogram of intraclass correlation coefficients across functional connections. For each connection, the intraclass correlation coefficient (ICC) with a two-way mixed-effects model (ICC3) was computed. Estimates ranged between -0.19 and 0.87 with a mean value of 0.19 and a positively skewed distribution.

E - The Neuroscience in Psychiatry Consortium

Principal investigators:

Edward Bullmore (CI from 01/01/2017)
Raymond Dolan
Ian Goodyer (CI until 01/01/2017)
Peter Fonagy
Peter Jones

NSPN funded staff:

Michael Moutoussis
Tobias Hauser
Sharon Neufeld
Rafael Romero-Garcia
Michelle St Clair
Petra Vértes
Kirstie Whitaker
Becky Inkster
Gita Prabhu
Cinly Ooi
Umar Toseeb
Barry Widmer
Junaid Bhatti
Laura Villis
Ayesha Alrumaithi
Sarah Birt
Aislinn Bowler
Kalia Cleridou
Hina Dadabhoy
Emma Davies
Ashlyn Firkins
Sian Granville
Elizabeth Harding
Alexandra Hopkins
Daniel Isaacs
Janchai King
Danae Kokorikou
Christina Maurice
Cleo McIntosh
Jessica Memarzia
Harriet Mills
Ciara O'Donnell
Sara Pantaleone
Jenny Scott

Affiliated scientists:

Pasco Fearon
John Suckling

Anne-Laura van Harmelen
Rogier Kievit
